# Reducing Time Taken to Gather Equipment for Venepuncture and Cannulation: A Quality Improvement Project

**DOI:** 10.7759/cureus.74285

**Published:** 2024-11-23

**Authors:** Luke Glover, Luna Nedic, Rebecca Myers, William Cuthbert, Ryan Smith, William Butterfield

**Affiliations:** 1 Medicine and Surgery, Royal Devon University Healthcare NHS Foundation Trust, Exeter, GBR

**Keywords:** cannulation, emergency, equipment, quality improvement, quality improvement projects, venepuncture

## Abstract

Introduction: Timely gathering of equipment for venepuncture or cannulation on hospital wards is important, particularly in emergency situations. Anecdotally several doctors working at a hospital in England expressed frustration at low equipment stock, layout, and discrepancies between wards leading to significant delays in this process. This quality improvement project therefore aimed to reduce the time taken to gather equipment for venepuncture or cannulation to 20 seconds by June 2023.

Methods: Quality improvement methodology was used to define the problem, produce an aim statement, and design several interventions. A flow map was created to understand the equipment collection process, a root cause analysis identified problem areas, and a driver diagram highlighted potential change ideas. A new trolley layout was implemented as part of several plan-do-study-act cycles with the addition of several simple human interventions to maximise its usage. More widespread introduction of identical trolleys across the surgical wards was also achieved.

Results: Initial qualitative surveys and the root cause analysis identified a lack of equipment availability, and discrepancies between wards being key barriers to rapid collection of equipment. Prior to any intervention, the average time taken to gather equipment for venepuncture and cannulation was 141 and 137.5 seconds respectively. After implementing a new trolley design and layout, with clear markings and human factor optimisation, the times were reduced to 18 seconds each. Subjective feedback during a cardiac arrest scenario following the intervention was positive. Widespread implementation of the trolleys around the hospital was started following this success although the efficacy of their introduction was not measured during the study period.

Conclusion: This quality improvement project successfully reduced the time taken to gather equipment for venepuncture or cannulation on a hospital ward, with positive feedback in an emergency. The project used well-documented quality improvement methodology to achieve this and highlights the ability of empowered clinical staff in non-managerial or non-leadership positions to action change.

## Introduction

Equipment gathering for basic procedures including venepuncture and cannulation can occupy a significant portion of a doctor’s time [1]. Delays in obtaining this equipment can significantly impact patient care, particularly in critical situations. In cardiac arrest or peri-arrest situations, rapid diagnostics such as blood gas analysis can help identify causes and guide management, while quick peripheral venous access enables life-saving interventions such as adrenaline administration [2,3]. 

However, doctors often work on-call and cover multiple wards; unfamiliar environments which have different layouts and mechanisms for provision of equipment. This project was inspired by frustrating experiences when trying to gather equipment at a hospital in England. In other hospitals, groups have had success in reducing this time-burden with optimisation of the equipment gathering process using quality improvement (QI) methodology [4-7]. 

Previous quality improvement projects based at the hospital had successfully introduced equipment trolleys onto some wards, however with no defined layout nor mechanism for regular restocking these were often empty and of minimal use. Ward stock separate to the trolley is also inconsistently laid out; one such example was blood bags being found on top of a cupboard in the sluice, an area typically reserved for disposal of effluence. 

Therefore, by identifying and addressing these layout and stocking barriers, this project aims to reduce the time taken to gather equipment for venepuncture or cannulation to 20 seconds by June 2023. To achieve this goal, objectives were set which included standardising equipment locations with a defined setup, implementing sustainable restocking mechanisms involving key stakeholders, and evaluating the impact of the interventions. If successful, the intervention could be expanded across wards to create a consistent and efficient system that ensures rapid access to critical supplies, ultimately enhancing patient care. 

## Materials and methods

**Figure 1:**
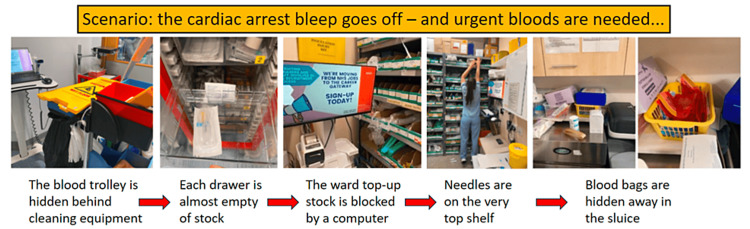
Pictorial Flow Map of the Process of Equipment Collection A pictorial flow map illustrating the process of collecting equipment for venepuncture and cannulation; particularly in emergency situations. In this case, the pre-existing ward blood trolley was found to be obstructed by cleaning equipment. Upon accessing the trolley, it was found to be virtually empty, so extra stock was needed from the ward top-up room. This stock was itself blocked by a ward workstation-on-wheels, and the butterfly needles could only be reached by the tallest of staff members. One amusing, if frustrating, finding was that of the blood bags – hidden away in the sluice. Image Credits: article authors.

As part of QI methodology [8], it was first important to understand the problem. A suitable ward was identified - a surgical ward which was known to have a stock trolley and would therefore provide a good base for tests of change. A pictorial flow map [9] of the process of equipment collection was taken to illustrate some of the frustrating barriers to timely collection in an emergency (Figure 1). 

**Figure 2:**
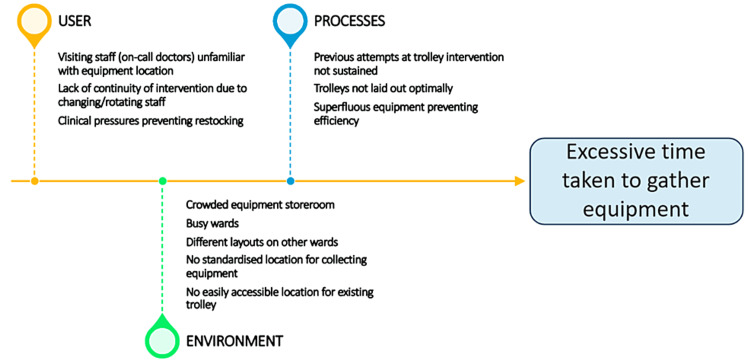
Root Cause Analysis A root cause analysis which explores some of the reasons behind excessive time taken to collect equipment for venepuncture or cannulation. These focussed on three main areas – user, processes and environment. In this case, a common theme was the lack of continuity or standardisation between different environments, compounded by the nature of work in the NHS – doctors are often transient members of the ward team. Image Credits: article authors.

A root cause analysis was then performed to understand the reasons behind the delay. This raised several possible factors, focussed largely on the environment and user; a clear theme was the lack of continuity and absent standardisation between different wards (Figure 2). 

The project then used the model for improvement to focus on the goal, measures and changes. The end goal was a reduction in time taken to gather necessary equipment for venepuncture or cannulation. A specific, measurable, achievable, relevant and time-based [10] aim was to reduce the time taken to gather equipment for venepuncture or cannulation to twenty seconds by June 2023. The measures were both objective and subjective; the former being time taken to gather equipment and the latter being feedback from stakeholders. Recognition of stakeholders was therefore of key importance and relevant people were quickly identified, including service users (primarily doctors and nurses), potential stockers (housekeeping staff and healthcare assistants) and other key players (ward matron, cluster manager and the hospital’s transformation team). These were all staff members who would be influential in achieving the aim and carrying any success forward in a sustainable way [11].

As part of the first Plan-Do-Study-Act (PDSA) cycle, the next step involved collecting measurements. Across several non-consecutive days, medical students and other volunteers were observed and timed collecting equipment for both venepuncture and cannulation using the existing ward set-up. Subjective feedback on the process of collection was also gathered. 

A survey containing seven questions was sent out to foundation doctors via different channels including email, social media group chats and advertisements in whole-year group lectures. The questions covered: current department, average time taken to gather equipment, experiences of well or poorly stocked wards, suggestion of change to current trolley which would increase efficiency, pieces of equipment that are particularly hard to come by and additional comments. The survey received 13 responses and, although not comprehensive, provided initial insights and identified further areas of development. 

**Figure 3:**
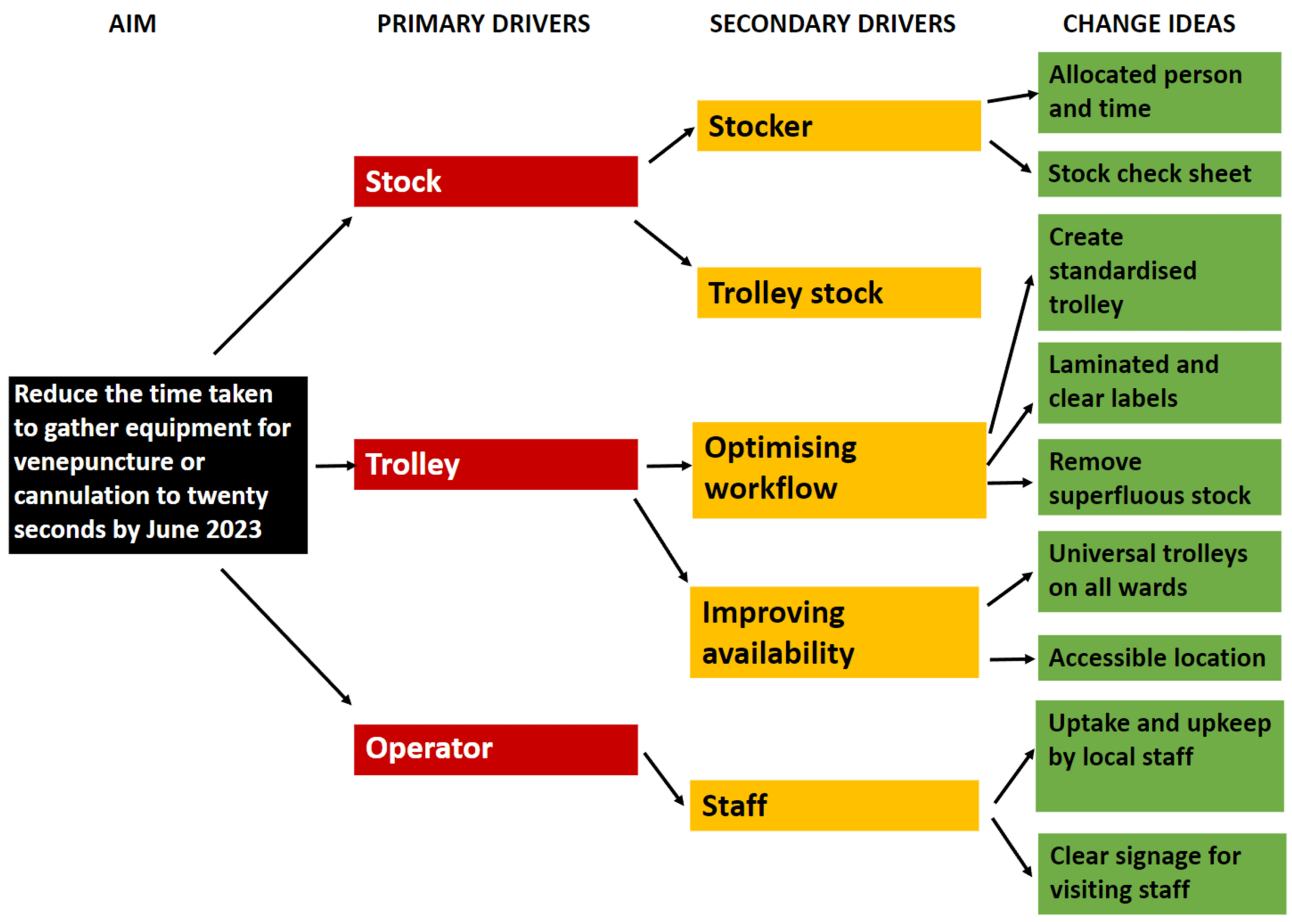
Driver Diagram A driver diagram exploring the broad factors which could influence the aim (primary drivers), the rough changes needed to influence these drivers (secondary drivers) and finally specific areas to target (change ideas). The driver diagram clarified which areas to target and gave several starting points for change. In a system that does not facilitate easy systems change, small interventions need to be powerfully targeted. The pre-existing equipment trolleys were a natural starting point for this. Image Credits: article authors.

After collection of baseline results, a driver diagram was created to explore factors that may lead to an improvement (Figure 3). 

**Figure 4:**
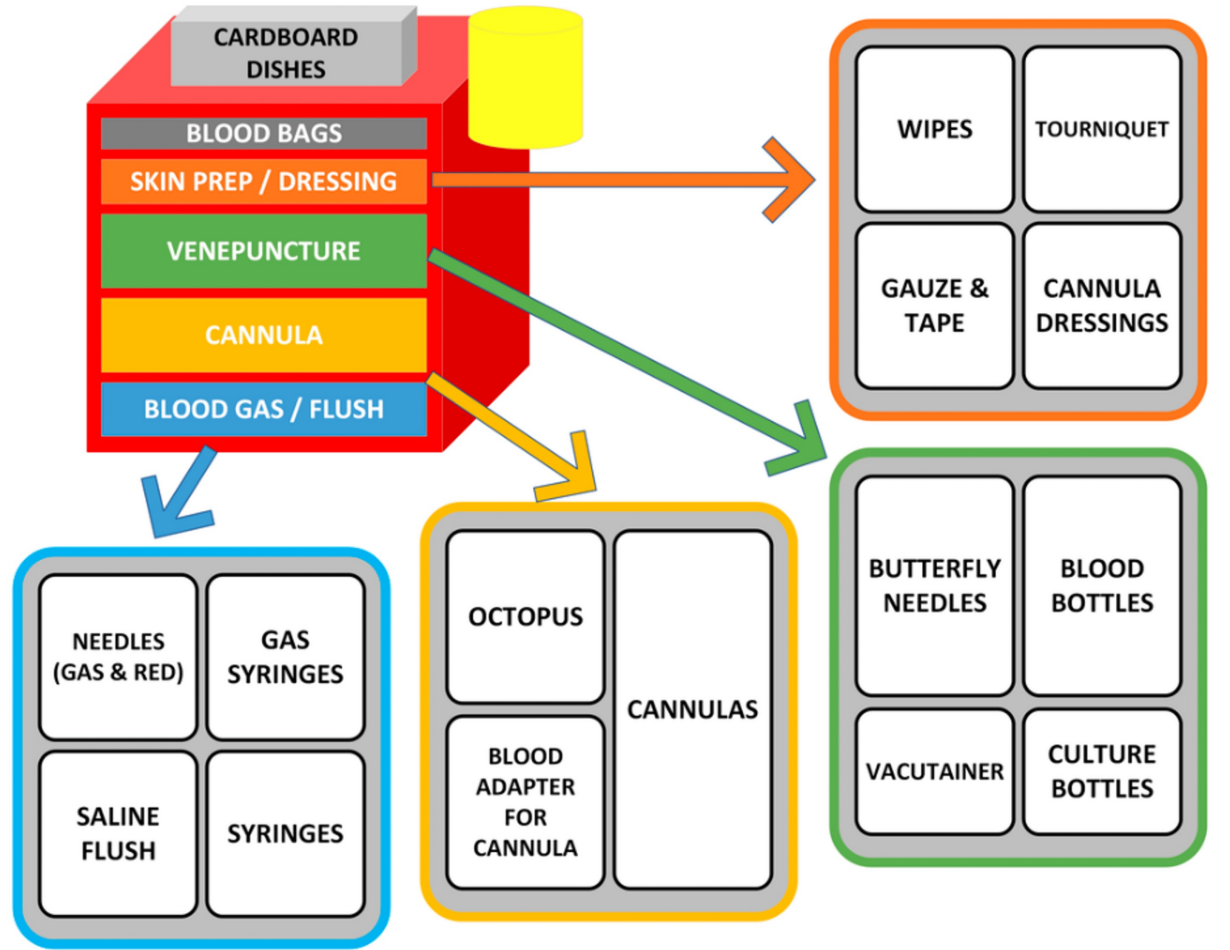
Schematic Diagram of New Trolley Layout A schematic diagram of a potential new, redesigned trolley layout, with thought given to the flow of equipment collection. Each drawer was designed to contain only the commonly used items, with superfluous equipment thought to affect stock and cause variable delay. On implementation of this design, it quickly became apparent that the existing trolleys had a fixed six drawer layout and so the design was quickly adapted. Image Credits: article authors.

**Figure 5:**
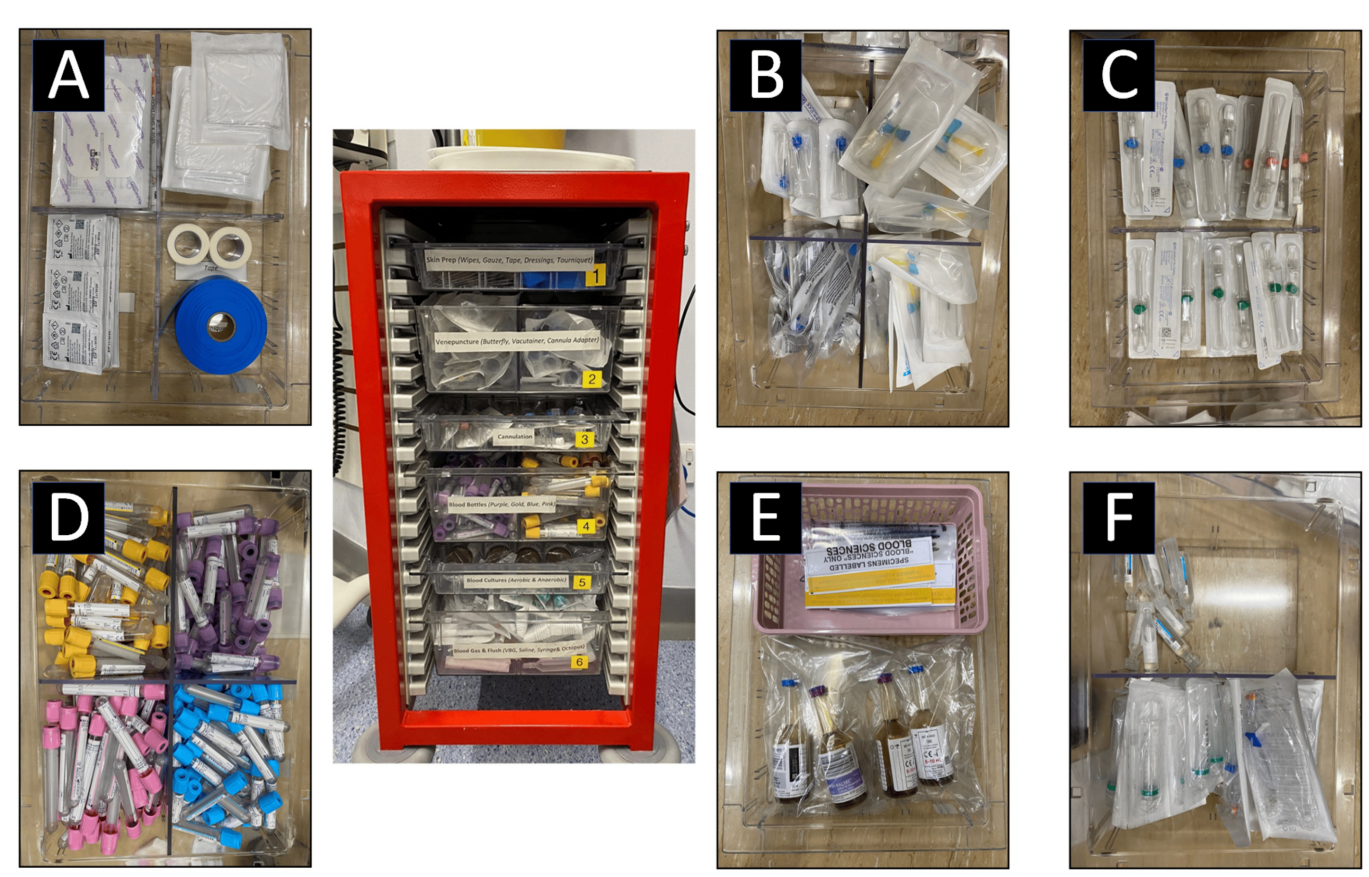
Newly Designed Trolley An image of the completed and redesigned equipment trolley with expanded views illustrating the layout and contents of each drawer from the top (labelled "A") through to the bottom (labelled "F") drawer. Each drawer is clearly labelled on the front with its contents. The user starts at the top with ”skin prep,” and works their way down, through drawers containing butterfly needles, cannulas, blood bottles, blood culture bottles, and flush paraphernalia respectively. Image Credits: article authors.

The second PDSA cycle utilised the results of the root cause analysis, measured objective data, subjective feedback and driver diagram. Optimisation and simplification of the existing trolley layout was hypothesised to be a relatively simple intervention that could result in significant improvement. As the chosen ward already had a trolley, photos of this were taken as a baseline for comparison. The project team then used their combined experience as well as trial and error to come up with a new layout (Figure 4). The goal was that rarely used, superfluous equipment could be removed, with remaining equipment then laid out in a more intuitive and end-process-driven way. The theoretical end-user could start at the top drawer (“Skin Prep”) and work their way down, collecting the equipment they need. Easily visible labels showing drawer contents would facilitate this. The eventual trolley layout, after several iterations, can be seen in Figure 5. 

A key element of the second PDSA cycle was the provision of a sustainable mechanism by which the trolley stock could be maintained. Initially this was by means of a “dedicated stocker;” a volunteer on the ward who would check stock in the trolley each day and replenish as necessary. Creation of a “stock check sheet” was hoped to facilitate and encourage this process by means of a daily check, akin to daily checks of resuscitation trolleys. 

After implementation of the new trolley and some indication of success, further study revealed that stocking the trolley was still problematic, and that the printed labels were not durable enough, frequently falling off and becoming worn out as the trolley was used. Subjective feedback showed visitors to the ward were not aware of the trolley’s existence nor its location. 

Therefore, as part of the third PDSA cycle, the labels were laminated as a more durable solution. A larger, laminated sign pointing to the defined location of the trolley was also implemented, primarily to aid unfamiliar or visiting staff. The most ambitious target was to spread the newly designed trolleys, with their associated environmental tweaks, to other wards around the hospital. Having shown their clear success, it was thought that this would be an easy feat; however, this proved challenging for a variety of reasons. Involvement of key stakeholders and early adopters (the leadership team on each ward, the hospital transformation team and divisional managers) facilitated this process. Initially, the surgical wards were assessed and four further wards which did not have any equipment trolleys were identified. Trolleys were purchased and successfully implemented across these wards. 

**Figure 6:**
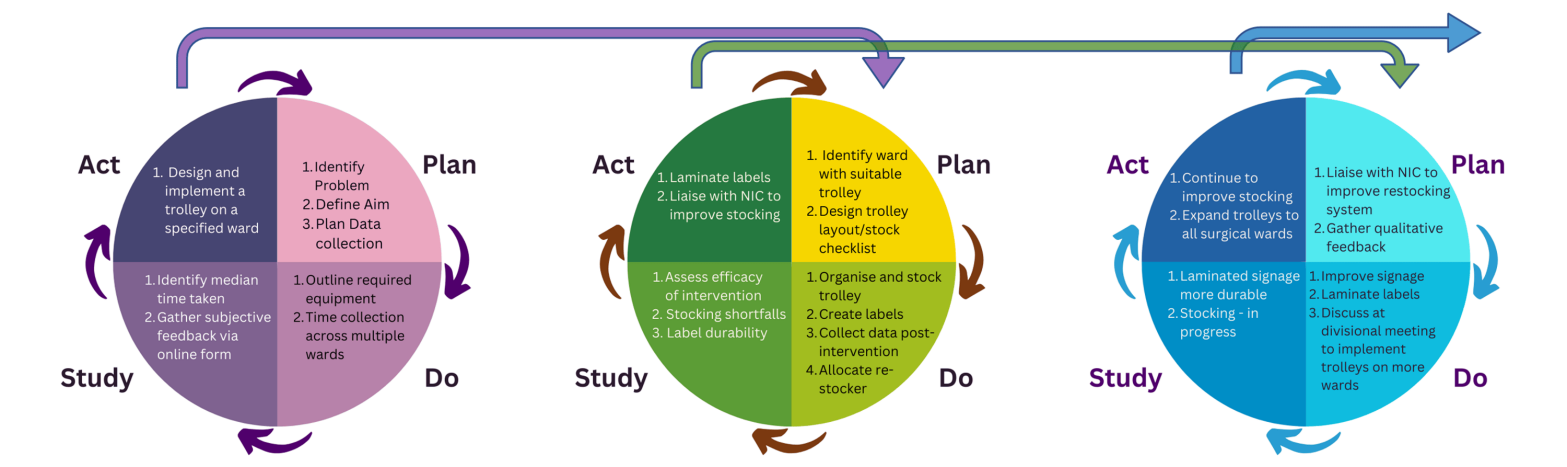
Plan-Do-Study-Act (PDSA) Cycles A graphical representation of the three PDSA cycles which made up much of the methodology of this quality improvement project. The first, left-most cycle focused on identifying the problem, defining aims, pre-intervention measurement and design of an intervention. The second, middle cycle implemented this intervention including liaison with the nurse in charge (NIC) as a test-of-change and studied its impact with objective and subjective data. This led to the third, right-most cycle which involved optimising the stocking of the trolley, improving durability of labels, and plans to implement the re-imagined trolley system across the wider hospital. Image Credits: article authors.

All three PDSA cycles are summarised in Figure 6. 

## Results

Initial surveys of doctors revealed significant discrepancies between wards and departments. Only some wards had equipment trolleys and amongst these, lack of standardised layout and contents was a common theme. Another prominent issue was stocking of the trolleys, and there were many comments about key items which were often missing (Table 1). 

**Table 1:** Pre-Intervention Survey Results Results from a pre-intervention survey sent out to doctors of varying experience who have worked across multiple wards, illustrating the key themes of variability between wards and departments, lack of standardisation, and poor stock as potential drivers for length of time taken to gather equipment for venepuncture and cannulation.

What changes could be made to the current blood trolleys to increase your efficiency at gathering the required equipment?	Are there any particular pieces of equipment you often have difficulty finding or are often in short supply?
Keep trolleys stocked	Butterfly needles, blood gas syringes, blood bottles
Same layout, easily identifiable colour	Blue cannula, gauze
Never any gas needles	Gas needles
Standardisation	Cannula dressings
Standardise them	Blood gas needles
Make them obvious, include in induction	Gauze
Easier to get drawers in/out as they stick	Butterfly needles and purple top blood bottles
Same place on every ward, same things in each drawer (e.g. cannula top drawer, blood bottles next drawer down)	Blood gas syringes
Keep them fully stocked	Flushes
Logical order, easy location of saline and syringe	Saline, syringe
Standard layout, lots of gauze, tape	Sharps bins. Gauze is in a different place everywhere.
Red trolley is easy to spot. The wards without trolleys are hard to navigate especially at night.	Blue butterfly needles, blood gas syringe

Pre-intervention data of 18 participants; 10 for venepuncture and eight for cannulation, was collected on the chosen ward. Results revealed a median time to gather equipment for venepuncture of 141 seconds and 137.5 seconds for cannulation. Subjective comments taken from participants generally revealed frustration and disbelief at the lack of stock, inefficient layout, and generally slow process. 

Following the introduction of the redesigned, optimised trolley with clear signage, the median time taken to collect equipment for venepuncture or cannulation was reduced to 18 seconds. Post-intervention feedback was also positive. One member of the ward staff gave anecdotal evidence of its success following a cardiac arrest on the ward, where the trolley was quickly wheeled over to facilitate rapid vascular access and blood gas sampling. A clinical matron described the trolley as “really useful.” Together, this feedback suggests that the improved trolley could have direct effects not only on user experience, but also patient safety. 

**Figure 7:**
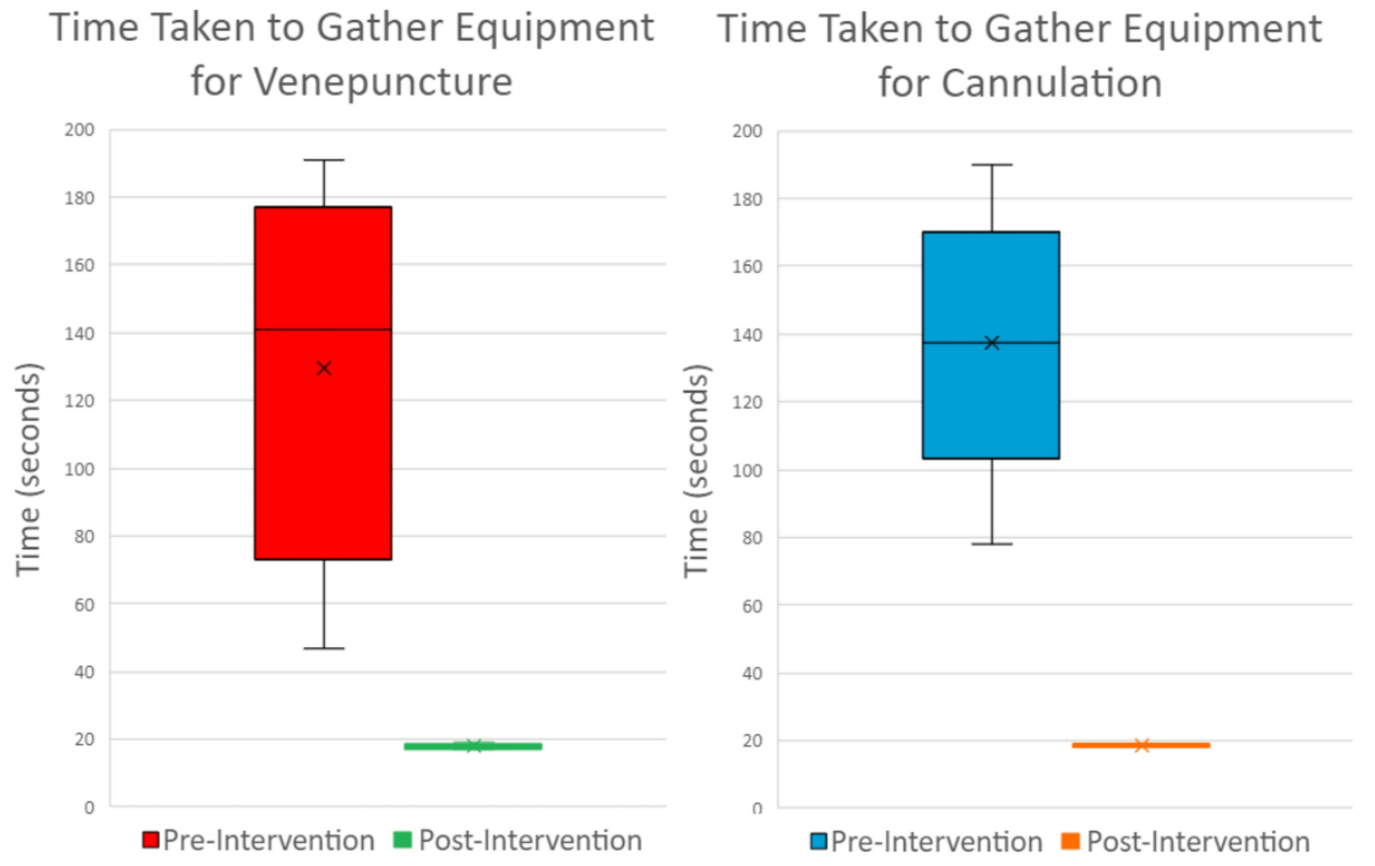
Pre- and Post-intervention Box-and-Whisker Plots Two box-and-whisker plots illustrating the time taken to gather equipment for venepuncture and cannulation both pre- and post-intervention. “X” represents the mean marker. Image Credits: article authors.

Pre- and post-intervention timings for both venepuncture and cannulation can be seen in Figure 7. 

Results on the efficacy of interventions to ensure regular stocking of the trolley were more mixed. The stock check-sheet was used sporadically, and a dedicated stocker was not found; high workload meant no one was able or willing to take up this role. Regardless, feedback from users was that staff would stock the trolley on an “ad-hoc” basis after using it. In the several months following implementation of the new trolley design, sporadic visits to the ward revealed a trolley that remained well-stocked and cared for, illustrating the value and sustainability of the changes made. 

Implementation of the trolleys throughout the wider hospital was more challenging, as it required liaison between multiple different departments, as well as agreement for adequate funding. Sharing the successes of the initial ward including the objective data and subjective feedback enabled recruitment of early adopters and key stakeholders including divisional managers, ward managers and the hospital transformation team, who could facilitate this process. After initially identifying four further surgical wards that had no equipment trolley, the cost of implementing this change was investigated and, after several months, the trolleys were implemented. 

## Discussion

This quality improvement project describes the use of established methodology to deal with a common issue; lack of equipment availability for venepuncture and cannulation, a problem which can affect patient safety, particularly in time-critical scenarios such as cardiac arrests. 

The first steps involved understanding the problem using a picture-based flow map, root cause analysis and gathering of subjective feedback. The model for improvement was then used to identify the goals, measures and changes that would be required to achieve the aim - a reduction in time taken to gather equipment for venepuncture or cannulation to 20 seconds by June 2023. To facilitate this, a driver diagram was created which gave some clear targets for interventions. Objective timings were taken pre-intervention as part of the PDSA cycles, and the intervention was then put in place - a complete redesign, restock and cultural shift in the use of the pre-existing stock trolley present on the chosen ward. The results of the intervention were studied again using objective timings, as well as subjective reviews of the restocking process and feedback from stakeholders including ward managers and end-users. Finally, a secondary aim of implementing the new trolleys across all surgical wards in the hospital was developed and QI methodology, including use of the “elevator pitch” and engagement with stakeholders and early adopters, was used to facilitate this change. 

Disseminating this improvement to the wider hospital was a significant challenge and illustrated the difficulties of implementing change on a larger scale. This provided several learning points. Despite the clear success and powerful feedback from users, there were not only logistical difficulties (it is difficult to make changes in environments in which one is not a permanent member) but also human factors, such as resistance to change. To this end, involvement of the senior ward team (key stakeholders - and potential “early adopters”) was an important step; these are staff members who lead by example, and with their enthusiasm the wider introduction of the new trolley was successful. The key importance that such individuals play cannot be underestimated. It also became apparent that in an overstretched system where staff have little free time and there is limited funding, it is vital to communicate a project in a compelling, effective way. 

A similar project in Bristol highlighted how robust data collection enhances negotiating strength when making a business case for hospital-wide change [7]. Although this project utilised both qualitative and quantitative baseline measurements similar to the current project - through a survey and a time trial, respectively - it assessed a greater number of data points, thereby providing stronger evidence to support their initiative. The article did not specify how these responses were obtained, making it difficult to directly compare why the current project’s survey participation was so limited. However, several possible barriers to obtaining responses from busy resident doctors were identified. These include perceived irrelevance, as the survey was distributed shortly after the August changeover, possibly leaving new resident doctors unable to identify with the issue sufficiently to comment; time constraints; lack of incentives; and poor communication or advertising. Another project with similar objectives and outcomes specifically compared datasets from equipment collected on a home ward versus an unfamiliar ward to demonstrate how familiarity affects efficiency and how standardisation can mitigate these delays-a critical point when seeking funding [4]. Although this project's data was not extensive, it was sufficient to demonstrate a clear improvement resulting from the changes implemented. However, careful consideration of data collection methods and alignment with the objectives of future projects is crucial, particularly when building a business case within an overstretched system like the NHS, where negotiating strength is essential. 

Finally, starting with a small change and assessing its effectiveness before expanding on a larger scale ensures sustainability and high motivation amongst the project team by valuing "small wins" [12]. 

This project had numerous limitations. The objective measurements were made by direct observation, and the Hawthorne effect (in its modern interpretation) could result in bias [13]. Indeed, the timers were part of the quality improvement project and could be vulnerable to observer bias. Following implementation of the new trolley layout, there was no formal collection of subjective data; which was instead gathered on an ad hoc basis and therefore will miss important positive or negative feedback. The most ambitious stage of the project (implementation of the trolleys throughout the wider hospital) was more challenging and though the first steps were achieved, the impact of the trolleys' introduction could not be measured during the study period. 

Going forwards, interventions to maximise the ongoing success of the project will be required, ensuring sustainable stocking of trolleys continues, with contemporaneous feedback and changes in response to this. Education of staff on the presence of trolleys will encourage engagement and maintenance, particularly when inducting new starters. Clear signage introduced by this project will also signpost non-ward staff (such as on-call doctors) to the trolley. Finally, the ultimate aim was the expansion of the trolley to acute wards hospital-wide with support of the hospital’s transformation teams, divisional cluster managers and clinical matrons. The sustainability of the original trolley intervention was encouraging, and it remains to be seen whether this success continues across the hospital. 

## Conclusions

Overall, this quality improvement project was a success, leading to a large reduction in time taken to collect equipment for venepuncture and cannulation on the chosen ward. Subjective feedback also revealed a direct effect on patient safety - rapid facilitation of venous access and blood taking in a cardiac arrest scenario, using the redesigned and optimised trolleys, helped by environmental changes to improve the trolley location, and systemic changes to highlight awareness. This project also began a further goal of disseminating the trolley to all surgical wards in the hospital, showing great engagement with key stakeholders and ability to enact systems change in unfamiliar environments. 

This project has illustrated the power of doctors and other healthcare workers, often transient members of a ward workforce, to improve patient safety through quality improvement. Simple, cheap and efficient “on-the-ground" interventions can be extremely effective and are often borne from knowledge that can only be gained by being a participant in the environments in which change is sought. 
